# The early onset of puberty and at the early onset of myopia: a cross-sectional evidence from girls at age 12 in Chongqing, China

**DOI:** 10.3389/fmed.2026.1728838

**Published:** 2026-03-25

**Authors:** Ruili Li, Jing Zhang, Yong Zhang, Tian OuYang, LiYan Gong, Dan Ao, Lan Tang, Li He, Xiaoya Qi, Yan Zuo, Qin Duan

**Affiliations:** 1 Department of Special Medical Service Centre, The First Affiliated Hospital of Chongqing Medical University, Chongqing, China; 2 Department of Health Management Center, The First Affiliated Hospital of Chongqing Medical University, Chongqing, China; 3 Health Medicine Center, The Second Affiliated Hospital of Chongqing Medical University, Chongqing, China; 4 School of Public Health, Chongqing Medical University, Chongqing, China

**Keywords:** girls, growth indicators, menarche age, myopia, puberty

## Abstract

**Introduction:**

The onset of menstruation, a pivotal marker of pubertal development, is recognized linking closely to the growth and health of females during adolescence and adulthood. The objective of this investigation is to elucidate the correlation between the early onset of menarche and the early onset of myopia.

**Methods:**

Female subjects aged 12 who took the enrollment physical examination required for junior high school admission were included. Eligible participants underwent anthropometric assessments, ocular examinations, and a structured questionnaire that included the age at menarche. Ocular parameters were compared between groups by menarche age. Univariate and multivariate logistic regression analysis were used to investigate the relationship between age at menarche and myopia in a sample of 468 participants, approximately 12 years of age.

**Results:**

The incidence of myopia in the right eye was observed at 82.7% within the early-menarche group (menarche age ≤ 11), succeeded by 75.8% in the normal-menarche group (menarche age > 11) and 72.3% in the late-menarche group (no menarche at age 12) (*p* = 0.081). For the left eye, the myopia prevalence was 77.0% in the early-menarche group, followed by 73.2% in the late-menarche group, and 67.3% in the normal-menarche group (*p* = 0.112). When moderate to severe myopia was used as the outcome, the early onset of menarche age was significantly and negatively related to myopia after adjusting for all possible confounders (*p* = 0.008 for the right eye, *p* = 0.004 for the left eye).

**Discussion:**

Our findings suggested that the early onset of puberty, indicated by early menarche, may trigger and exacerbate the progression of myopia in early adolescence. Additional interventions may be needed to prevent the early onset of myopia at the early stage of adolescence.

## Introduction

Myopia has emerged as a global public health challenge, with its incidence rate experiencing a dramatic upsurge (1), subsequently imposing substantial economic burdens (2). Notably, East and Southeast Asia exhibit a pronounced epidemiological prevalence among children, and the phenomenon of myopia at an earlier age has garnered close attention from researchers and government departments in recent years (3).

The onset of menstruation, a crucial milestone of female maturation, serves as an indicator for assessing the progression of pubertal development (4). Recent data reveal a decreasing trend in the age of menarche among Chinese females, with the median age at menarche declining from 13.37 years in 1985 to 12.00 years in 2019 (5). Current research yielded inconsistent results about menarche and myopia in girls. Some studies have established a correlation (6–10), while others assert a limited association (11–13), and yet more indicate no significant association (14, 15).

This inconsistency partly stems from the imprecision in defining myopia (6–8, 12), as well as the methodological limitations associated with recall bias (9, 10, 13). The year of conducting the studies may be one possible reason for the inconsistency, as lifestyle changes dramatically over time. Moreover, because age is an important factor in myopia in children, older children tend to have a higher prevalence of myopia. When sample ages in existing studies vary, it may lead to inconsistencies in findings among studies.

Based on the extant literature, we know that myopia is continuously soaring up and exacerbating short eyesight in children. The relationships between menarche and myopia at the early stage of puberty may provide us with a jigsaw for better understanding the dynamic development of myopia in children and developing the intervention and control strategies targeting myopia. However, with a paucity of research focused on 12-year-old girls, the evidence regarding the relationship between menarche and myopia in 12-year-old girls remains insufficient.

## Methods

### Study participants

The subjects were female primary school graduates from the Chongqing urban area who came to the hospital for a physical examination to enroll in junior high school. They were around 12 years old at that age. The Ethics Committee of Chongqing Medical University obtained ethical approval (No. CAF52704054B). The purposes and contents of the study were explained to all subjects and their parents or legal guardians, and written consent was obtained from all subjects involved.

Students who refused to participate or had a medical history of eye diseases such as strabismus, amblyopia, ocular inflammation, ocular trauma, corneal disease, congenital cataract, choroid or retinal disorders, and high astigmatism (astigmatism greater than three diopters or anisometropia greater than three diopters) and high hyperopia (hyperopia more than three diopters), and those had been using atropine eye drops, red-light therapy or immunoglobulin heavy chain (IGH) treatment, which may influence the onset of myopia, were not eligible to participate in the study.

### Ocular examinations

Ocular examinations containing visual acuity, slit-lamp examination, direct ophthalmoscopy, and non-cycloplegic refraction. In this study, measurements were taken of both eyes. All subjects underwent measurement of uncorrected distance visual acuity (UCDVA) at 5 m (standard logarithmic visual acuity E chart) and recorded in Log MAR scores. Visual acuity was tested both with and without refractive correction for those wearing spectacles.

Although the gold standard method to myopia detection is cycloplegia refraction, many previous studies have convinced that there is no difference in children’s accurate refraction measured by non-cycloplegic or cycloplegic measurement when children beyond 10 years old (16), refraction was obtained by non-cycloplegia and no fogging measurement using the Automatic Computerized Refractometer (HRK-7000A, Huvitz Co. Ltd.). Both eyes of each subject were measured at least thrice in a darkened room. If the difference of refractive error (RE) between measurements reached 0.50 diopters (D) or above, additional examinations were conducted. The mean values of spherical diameter (SD) and cylindrical diameter (CD) were used for analysis.

### Physical examination

Physicians from the Department of Health Medical Center conducted physical examinations by the “2014 National Physical Fitness and Health Survey Rules” (17), which included measurements of height (in meters), weight (in kilograms), heart rate (in beats per minute), and blood pressure (in millimeters of mercury). The Body Mass Index (BMI) was calculated as body weight (in kilograms) divided by height (in meters) squared.

### Pulmonary function test (PFT)

We included pulmonary function tests (PFT) as an indirect measure of students’ physical activity levels (18). Pulmonary Function equipment (COSMED) was used to measure students’ PFT. The measurement values of Forced Vital Capacity (FVC), Forced Expiratory Volume in one second (FEV1), FVC% (the percentage of actual FVC compared to predicted FVC based on age, gender, and height), FEV1% (the percentage of actual FEV1 compared to predicted FEV1 based on age, gender, and height) were recorded and were used for analysis.

### Questionnaire survey

A questionnaire was administered in face-to-face interviews by doctors to collect information, including the age of menarche, demographic and status, myopia of parents, and behavioral information about eyesight and the academic performance (the prestige of the school to which they were admitted). To avoid the biases of researchers, a fixed team administered all questionnaires.

### Definitions

Uncorrected visual acuity (UCVA) of ≤0 LogMAR was considered normal vision. The spherical equivalent refraction (SER) was converted by adding the spherical refraction and half the cylindrical refraction. Myopia was defined as UCVA > 0 log MAR in either eye with SER ≤ −0.50 D (18). The myopia was divided into three levels according to SER: mild myopia −3.0 D < SER ≤ −0.5 D; moderate myopia −6.0 D < SER ≤ −3.0 D; severe myopia SER ≤ −6.00D (19).

In this study, the year of menarche was self-reported by the students. Menarche status was divided into three groups arbitrarily in this study: female subjects experiencing menarche at the age of ≤ 11 were classified into the early-menarche group, and those with an onset of menarche at the age of >11 were allocated to the normal-menarche group. In contrast, individuals who had not yet undergone menarche by the time of the survey were termed as the late-menarche group.

### Quality control

Before the study started, all members of the research team, including three experienced ophthalmologists, two qualified optometrists, and one post-graduate, were trained. All instruments were checked and adjusted before the examination. EpiData 3.1 was used for data double entry.

### Data analysis

Data were summarized as proportions for categorical variables or as mean ± standard deviation (SD) for continuous variables. Variables were compared using Chi-square tests or Analysis of Variance (ANOVA) among the three menarche groups. The association between menarche and myopia was analyzed using univariate or multivariate logistic regression. A two-sided *p* value < 0.05 was considered statistically significant. All statistical analyses were conducted using SPSS 19.0.

## Results

### General characteristics of participants divided by menarche status

Within a sample population of 468 participants, three distinct groups were identified based on the timing of menarche: 191 girls with menarche age ≤ 11 were assigned to the early-menarche group, 165 girls with menarche > 11 to the normal-menarche group, and 112 girls with no menarche to the late-menarche group. The characteristics of the 468 enrolled subjects, categorized by whether they experienced menarche or not, were summarized in Table 1. Early menarche was associated with higher height, weight, BMI, SBP, and PET. There were no significant differences between the three groups in the myopic condition of parents, academic performance (the prestige of the schools they were admitted to), heartbeat rate, and DBP.

**Table 1:** Characteristics of the study population according to menarche age.

Variable	Early-menarche	Normal-menarche	Late-menarche	*p*
	*N* = 191	*N* = 165	*N* = 112	
Age (year)	11.93 ± 0.25	11.98 ± 0.15	11.92 ± 0.27	0.084
Height (cm)	158.45 ± 5.62	157.83 ± 5.27	153.69 ± 6.34	<0.001
Weight (Kg)	51.53 ± 9.7	47.62 ± 7.36	40.5 ± 8.39	<0.001
BMI (Kg/m^2^)	20.46 ± 3.29	19.1 ± 2.74	17.08 ± 2.98	<0.001
Parents myopia				
Father (*n*, %)	75 (39.3)	61 (37)	43 (38.4)	0.905
Mother (*n*, %)	82 (42.9)	81 (49.1)	47 (42.0)	0.394
Father and mother (*n*, %)	45 (23.6)	35 (21.2)	22 (19.6)	0.709
Academic performance				0.383
Low (*n*, %)	23 (12.0)	15 (9.1)	8 (7.1)	
Middle (*n*, %)	126 (66.0)	102 (61.8)	72 (64.3)	
High (*n*, %)	42 (22.0)	48 (29.1)	32 (28.6)	
Heartbeat rate (time/min)	80.81 ± 12.31	80.16 ± 12.76	80.08 ± 11.65	0.841
Blood pressure				
SBP (mmHg)	107.32 ± 12	106.24 ± 10.28	103.08 ± 10.58	0.005
DBP (mmHg)	63.87 ± 8.71	64.27 ± 7.42	63.43 ± 6.84	0.678
PET				
FVC (liter)	2.71 ± 0.4	2.65 ± 0.39	2.39 ± 0.41	<0.001
FVC%	90.67 ± 11.62	88.97 ± 12.4	85.83 ± 11.88	0.006
FEV1 (liter)	2.47 ± 0.48	2.36 ± 0.34	2.15 ± 0.38	<0.001
FEV1%	88.02 ± 10.87	85.76 ± 10.65	83.23 ± 11.8	0.002

Early-menarche: menarche age ≤11; normal-menarche: menarche age>11; Late-menarche: have not experienced menarche.

PFT, Pulmonary Function Test; FVC, Forced Vital Capacity; FEV1, Forced Expiratory Volume in one second; FVC%, the percentage of actual FVC; FEV1%, the percentage of actual FEV1 compared to predicted FEV1.

### Ocular parameter compared by menarche age

Table 2 revealed that earlier menarche group exhibited significantly reduced spherical diameter (SD) and spherical equivalent refraction (SER) in both ocular regions. Additionally, the three groups demonstrated statistically significant variations in uncorrected visual acuity (logMAR) in their left eyes. Notably, no significant differences in cylindrical diameter (CD) were observed across the groups.

**Table 2:** Ocular parameter of participants compared by menarche age.

Variable	Early-menarche	Normal-menarche	Late-menarche	*p*
	*N* = 191	*N* = 165	*N* = 112	
Right eye				
logMAR*	−0.46 ± 0.35	−0.38 ± 0.32	−0.41 ± 0.34	0.065
SD	−1.99 ± 1.89	−1.51 ± 1.97	−1.44 ± 1.94	0.020
CD	−0.88 ± 0.72	−0.73 ± 0.75	−0.76 ± 0.79	0.121
SER	−2.4 ± 1.93	−1.86 ± 2.02	−1.8 ± 2	0.011
Left eye				
logMAR*	−0.45 ± 0.34	−0.33 ± 0.31	−0.39 ± 0.33	0.003
SD	−1.69 ± 1.97	−1.11 ± 1.79	−1.23 ± 1.74	0.009
CD	−1.12 ± 0.75	−0.99 ± 0.81	−1.06 ± 1.53	0.501
SER	−2.22 ± 2.04	−1.6 ± 1.9	−1.74 ± 2.09	0.010

Early-menarche: menarche age ≤11; normal-menarche: menarche age>11; Late-menarche: have not experienced menarche.

SD, the spherical diameter; CD, the cylindrical diameter; SER, the spherical equivalent refraction.

^*^Uncorrected visual acuity was used.

### Myopia prevalence by menarche status

The data presented in Table 3 indicated that the incidence of myopia in the right eye was observed at 82.7% within the early-menarche cohort, succeeded by 75.8% in the normal-menarche cohort and 72.3% in the late-menarche group. For the left eye, the peak myopia prevalence was 77.0% in the early-menarche group, trailed by 73.2% in the late-menarche group and 67.3% in the normal-menarche group. Statistical analysis revealed no significant discrepancies in myopia prevalence according to menarche age (*p* = 0.081 for the right eye, *p* = 0.112 for the left eye). Similarly, no statistically significant differences were detected in the severity of myopia about the age of menarche for either eye (*p* = 0.102 for the right eye, *p* = 0.062 for the left eye).

**Table 3:** Comparison of the myopia* prevalence between difference menarche age groups.

Variable	Early-menarche	Normal-menarche	Late-menarche	*p*
	*N* = 191	*N* = 165	*N* = 112	
Right eye				
Myopia				0.081
No	33 (17.3)	40 (24.2)	31 (27.7)	
Yes	158 (82.7)	125 (75.8)	81 (72.3)	
Myopia severity				0.102
No	33 (17.3)	40 (24.2)	31 (27.7)	
Mild	84 (44.0)	83 (50.3)	50 (44.6)	
Moderate	68 (35.6)	38 (23.0)	29 (25.9)	
Severe	6 (3.1)	4 (2.4)	2 (1.8)	
Left eye				
Myopia				0.122
No	44 (23.0)	54 (32.7)	30 (26.8)	
Yes	147 (77.0)	111 (67.3)	82 (73.2)	
Myopia severity				0.062
No	44 (23.0)	54 (32.7)	30 (26.8)	
Mild	79 (41.4)	76 (46.1)	56 (50.0)	
Moderate	59 (30.9)	30 (18.2)	22 (19.6)	
Severe	9 (4.7)	5 (3.0)	4 (3.6)	

Early-menarche: menarche age ≤11; normal-menarche: menarche age>11; Late-menarche: have not experienced menarche.

*Uncorrected visual acuity was used.

### Logistic regression analysis of myopia and menarche age

Table 4 showed the results of Univariate and multivariate logistic regression between menarche age and myopia. It revealed that among 12-year-old girls, an earlier age at menarche was inversely associated with the risk of myopia in the right eye (*p* < 0.05). Following adjustment for potential confounders, the correlation between menarche age and myopia risk in the right eye retained partial significance (*p* < 0.05). Girls who experienced early menarche exhibited a higher propensity for myopia compared to those with normal or late menarche.

**Table 4:** Logistic regression analysis of myopia and menarche status.

Variable	Myopia vs. normal (Right eye)			Myopia vs. normal (left eye)		
	*β*	SE	*p*	*β*	SE	*p*
Model 1	−0.306	0.149	0.04	−0.142	0.139	0.309
Model 2	−0.324	0.166	0.05	−0.197	0.155	0.203
Model 3	−0.366	0.171	0.032	−0.248	0.161	0.124
Model 4	−0.371	0.172	0.032	−0.237	0.163	0.146

Menarche status was coded with 1, 2, 3 for early menarche, normal-menarche, and late-menarche, respectively.

Model 1, without any adjusting.

Model 2, adjust by BMI, Heart beat rate, SBP, DBP.

Model 3, Model 2 plus FVC, FVC%, FEV1, FEV1%.

Model 4, Model 3 plus father myopia, mother myopia and academic performance.

### Logistic regression analysis of moderate/severe myopia and menarche age

Upon evaluating the severity of myopia, we conducted a Univariate and multivariate logistic regression to examine the association between the age of menarche and the risk of moderate to severe myopia. As depicted in Table 5, the age of menarche exhibited a significant negative correlation with the risk of moderate to severe myopia in both eyes (both *p* < 0.05), even after adjusting for potential confounders. Pre-adolescent girls aged 12 with early menarche demonstrated a notably heightened risk of moderate to severe myopia compared to their counterparts with regular or delayed onset of menarche.

**Table 5:** Logistic regression analysis of moderate/severe myopia and menarche status.

Variable	Moderate/severe vs. normal/mild myopia (Right eye)			Moderate/severe vs. mild myopia/normal (left eye)		
	*β*	SE	*p*	*β*	SE	*p*
Model 1	−0.327	0.137	0.017	−0.394	0.144	0.006
Model 2	−0.424	0.152	0.005	−0.492	0.159	0.002
Model 3	−0.431	0.155	0.005	−0.474	0.162	0.003
Model 4	−0.418	0.158	0.008	−0.467	0.164	0.004

Menarche status was coded with 1, 2, 3 for early menarche, normal-menarche, and late-menarche, respectively.

Model 1, without any adjusting.

Model 2, adjust by BMI, Heart beat rate, SBP, DBP.

Model 3, Model 2 plus FVC, FVC%, FEV1, FEV1%.

Model 4, Model 3 plus father myopia, mother myopia and academic performance.

## Discussion

Our investigation revealed that, even after adjusting for variables related to the physical development of girls at an early stage of puberty, such as body BMI, heart rate, SBP, and DBP, lung functions, or variables related to genetic factors such as parents myopia or variable related to the eye using habit such as academic performance, the age of menarche exhibited a significant and negative relationship to myopia in the right eye (*p* = 0.032). This may indicate that menarche, as a marker of the onset of puberty, can be an independent risk factor for myopia. However, this relationship was not significant in the left eye (*p* = 0.146). This may indicate that the two eyes were not synchronous in the onset or development of myopia. The onset or progression of myopia may be delayed due to different eye-using habits between the right and left eyes.

We, therefore, further investigated the influence of menarche on the occurrence of moderate to high myopia in both eyes. It was interesting that menarche was significantly was significantly related to both eyes negatively in severe myopia (right eye, *p* = 0.005; left eye, *p* = 0.002). Because time is needed for newly emerging myopia to progress into moderate or severe myopia, only early-emerging myopia has a chance to become moderate or severe myopia. Therefore, the outcome of moderate to severe myopia in this study may reflect the early onset of myopia under certain conditions, such as early puberty or poor eye-use habits.

Besides our data, the phenomenon that the right eye is more susceptible to myopia than the left eye has been found by others (20). This difference in susceptibility to myopia between the two eyes may be one of the reasons why only a relationship between myopia and menarche in the right eye was found at age 12. At the early stage of puberty, the onset of myopia in the left eye may not be sufficient to be detected. However, it can be detected with age, as was evident in one of our prior study (21), which included girls aged 15.

Our study suggests that menarche age, as an indicator of puberty, is closely related to the onset of myopia, which is also verified by other indicators of puberty in studies, which found that a notable positive correlation between myopia and children’s physical measurements, such as weight and height (22–25).

Because myopia onset is at the start of puberty and continuously worsens after that, myopia prevalence is dynamic progress in puberty or adolescence period. Investigations at different timing points may yield different results. For example, investigations by Nirmalan et al. (9) and Lyu et al. (10, 13) involving adult cohorts revealed an inverse and significant relationship between age at menarche and myopia. However, research by Wu (12) in 2014, focusing on female subjects aged 9–13 in Anhui Province, China, revealed no significant differences in the prevalence of myopia between the premenstrual and postmenstrual girls without considering the specific ages of menarche. In Karen’s (14) study, the subjects’ ages were lower than 17, and in Yip’s (15) study, the subjects’ ages ranged from 6 to 14. Both studies included subjects with heterogeneity in age. Moreover, a similar situation was observed in the study by Wang et al. (11), which included girls aged 7–13 from 2015 to 2017. Age can be a potent confounder, distorting the relationships between menarche and myopia.

Besides the confounder of age, other reasons may contribute to inconsistent conclusions among studies, such as variations in the definition of myopia. For example, in Li′s (6) and Xu’s investigations (7, 8), myopia is determined solely by the uncorrected distance visual acuity. Furthermore, these studies were conducted before 2014, a decade ago. After that, there has been a significant early shift of the pubertal age worldwide (5).

Regarding the mechanisms by which menarche is related to myopia, sex hormones, particularly estradiol, may play an important role (26). Estradiol influences the progression of myopia by modulating various intraocular enzymes (27) and corneal characteristics (28). Moreover, variations in the levels of insulin-like growth factor-1 may underpin the molecular basis of the association between myopia and the age of menarche (29). One of our previous study (30) has also indicated that a spurt of height growth due to puberty accelerates the process of emmetropization.

### Strengths and limitations

This study possesses several notable strengths. First, the participants exhibited a high degree of homogeneity in age. They shared everyday living and educational environments, facilitating the exclusion of potential confounders, such as age and behaviors, from the analysis. Second, the utilization of spherical equivalent refraction (SER) measurements enhances the precision of myopia detection over traditional vision acuity chart tests or questionnaires, which are commonly employed in large-scale studies. Third, data collection through face-to-face interviews is likely to yield more reliable information regarding the age of menarche compared to self-administered questionnaires.

### Limitations

However, it is imperative to acknowledge the limitations inherent in the present study. Initially, the sample size is relatively modest, originating from a single site, which introduces selection bias and precludes generalizability. Second, the retrospective cohort design of our research suggested a causal relationship between menarche and myopia. However, recall bias of data collection may exist. A prospective study might provide greater clarity on the causal nexus between them. Third, the absence of hormonal level data, the gold standard for assessing puberty, leaves the mechanism linking menarche to myopia unresolved. Lastly, the unavailability of potential confounders, such as sociological status, lifestyle behaviors, namely eye usage habits, outdoor activity, screen time, and sleep quality —may have skewed the results.

## Conclusion

Myopia in early adolescence is significant at the onset of puberty. The implementation of additional proactive intervention should be developed to prevent myopia due to the early onset of puberty. Future research should be conducted to delve into the mechanisms and patterns of myopia development in those girls with early onset of puberty.

## Data Availability

The raw data supporting the conclusions of this article will be made available by the authors, without undue reservation.
